# Estrés y depresión en el entorno académico-social durante el 2020 en estudiantes de odontología

**DOI:** 10.21142/2523-2754-0904-2021-080

**Published:** 2021-12-09

**Authors:** Francis Valeria Espinoza Vilela, Carol Magaly Cárdenas Flores

**Affiliations:** 1 Carrera de Estomatología, Universidad Científica del Sur. Lima, Perú. francisespinoza030798@gmail.com, carytocf@gmail.com Universidad Científica del Sur Carrera de Estomatología Universidad Científica del Sur Lima Peru francisespinoza030798@gmail.com carytocf@gmail.com

**Keywords:** estrés, depresión, COVID-19, estudiantes, odontología, stress, depression, COVID-19, students, dentistry

## Abstract

**Objetivo:**

La nueva modalidad de clases virtuales que reciben los estudiantes de odontología puede traer efectos negativos para la salud psicológica o el desempeño académico. El objetivo fue determinar la relación del estrés y depresión en el entorno académico social durante el 2020 en los estudiantes de Odontología de la Universidad Científica del Sur.

**Materiales y métodos:**

El estudio fue de tipo descriptivo observacional, transversal y prospectivo, en el que participaron 108 estudiantes de la carrera de Odontología de 6.^o^ a 10.^o^ ciclo de la Universidad Científica del Sur. Los participantes fueron evaluados mediante un cuestionario con la escala SISCO para medir el estrés académico y la escala BECK (IDB), que se utilizó para evaluar la depresión.

**Resultados:**

En el 6.^o^ ciclo existe probabilidad de estrés y depresión; el grupo etario más afectado fueron los estudiantes de 19 a 22 años. El valor p para la relación entre el estrés académico y depresión fue p < 0,001

**Conclusión:**

Existen diferencias estadísticamente significativas entre el estrés académico y la depresión en los estudiantes del 6.^o^ al 10.^o^ ciclo de la carrera de Estomatología de la Universidad Científica del Sur.

## Abstract (en)

**Aim:**

The new modality of virtual classes undertaken by dental students can have negative effects on psychological health and / or academic performance. The objective of this study was to determine the relationship between stress and depression in the social academic environment in dentistry students at the Científica del Sur University in 2020.

**Materials and Methods:**

The study was descriptive, observational, cross-sectional, and prospective. 108 students from 6th to 10th cycle of Dentistry at the *Universidad Científica del Sur* participated and were evaluated by means of a questionnaire with the SISCO Inventory to measure academic stress. The BECK scale (BID II) was also used for the evaluation of depression.

**Results:**

Students in the 6th term presented a greater probability of stress and depression. The age group most affected was students between 19 and 22 years of age. The p value for the relationship between academic stress and depression was p <0.001

**Conclusion:**

There are statistically significant differences between academic stress and depression in students from the 6th to the 10th term of the Stomatology career at the *Universidad Científica del Sur.*

## INTRODUCCIÓN

Debido a la COVID-19, la población peruana viene atravesando cuadros de estrés y depresión por el nuevo estilo de vida y restricciones que plantea el Estado. Uno de los cambios más radicales ha sido implementar la modalidad de enseñanza virtual en colegios, institutos y universidades ^1^. La carrera de Odontología se caracteriza por ser exigente y de ámbito teórico-práctico, ya que en los últimos años de carrera se realizan tratamientos en pacientes. Bajo la nueva modalidad, los estudiantes de dicha carrera vienen afrontando clases 100% teóricas, pese a la necesidad de tener horas prácticas. Este factor, así como la sobrecarga de trabajos, tareas, exámenes y clase de horas prolongadas, pueden afectar la salud psicológica o el desempeño en sus clases ^2^^-^^4^.

Es probable que los trabajos, exámenes y horas prolongadas de clases virtuales estén preocupando y demandando mucho tiempo semanal de los estudiantes, lo que influye en su estado anímico y desenvolvimiento en las clases ya que, a pesar de estar en casa todos los días, invierten más de 40 horas en promedio de clases teóricas semanales, las cuales pueden abarcar de 8-10 horas diarias sin tomar en cuenta el tiempo que utilizan realizando trabajos y tareas fuera del horario curricular ^5^^-^^7^. Esta carga académica se viene manifestando en los estudiantes con varios síntomas que conllevan al estrés y la depresión, ya que bajo el aislamiento social no les permite despejarse ni los domingos, a pesar de estar seis días de la semana dedicados la mayor parte del tiempo a los estudios ^8^^-^^10^.

Ante esta situación, es muy importante tener estrategias que permitan reducir los niveles de estrés y depresión que aparecen en la vida de los universitarios. El objetivo del estudio fue determinar la relación del estrés y la depresión con el entorno académico-social durante el 2020 en los estudiantes de Odontología de la Universidad Científica del Sur.

## MATERIALES Y MÉTODOS

El estudio fue de tipo descriptivo-observacional, prospectivo y transversal. La población de estudio comprendió a los estudiantes de la carrera de Estomatología de la Universidad Científica del Sur. La muestra se realizó mediante la fórmula de proporción Fisterra. Se incluyó a los estudiantes de manera aleatoria, sin antecedentes de enfermedades psicológicas, que cursaban del 6.^o^ al 10.^o^ ciclo de la carrera durante el 2020, y que decidieron participar en el estudio y firmaron el consentimiento informado. Se les aplicó dos instrumento de evaluación mediante el uso de dos cuestionarios validados en el país, escala SISCO y escala de Beck.

### Instrumento de Evaluación Estrés SISCO

El cuestionario SISCO consta de 31 preguntas objetivas que permiten determinar en qué categoría se encontraron los participantes, ya sea bajo (0-79), medio (80-88), medio (88) o alto (89-180), mediante la sumatoria e interpretación según la respuesta del participante, teniendo en cuenta una escala del 1 al 5, en la que 1 es Nunca, 2 es Rara vez, 3 es Algunas veces, 4 es Casi siempre y 5 es Siempre.

### Instrumento de Evaluación Depresión Beck (IDB)

El cuestionario IDB consta de 21 preguntas objetivas que permiten determinar en qué categoría se encuentran los participantes: Altibajos considerados normales (0-10), Leve perturbación del estado de ánimo (11-16), Estados de depresión intermitentes (17-20), Depresión moderada (21-30) o Depresión grave (31-40), una vez completado el cuestionario.

Se sumaron los puntos correspondientes a cada una de las 21 preguntas y se obtuvo el total con la finalidad de medir tanto el estrés académico como la depresión en los alumnos que cursaban del 6.^o^ al 10.^o^ ciclo de la carrera. El investigador se calibró en el empleo de los cuestionarios indicados y se realizó la prueba piloto al 10% de la muestra que indican los antecedentes con un Kappa = 0,85 para hallar el tamaño de muestra se utilizó el estimador de tamaño muestral de proporción Fisterra N = 108.

La investigación fue evaluada por el Comité de Ética de la Universidad Científica del Sur, los correos electrónicos proporcionados serán confidenciales y no serán utilizados para otros fines que no sean parte de la investigación, a fin de proteger la confidencialidad del participante.

## RESULTADOS

El plan estadístico se realizó utilizando el sistema SPSS (Inc Armonk NY, EE. UU.) para evaluar las variables estrés académico y depresión en estudiantes de odontología de 6.^o^ a 10.^o^ ciclo de la Universidad Científica del Sur. La muestra estuvo compuesta por 108 alumnos, divididos en 3 grupos etarios: 19-22 años, 23-25 años y 26 años a más. 

Se evaluó la relación de estrés (escala SISCO) y el grupo etario, y se encontró estrés alto en el grupo de 19 a 22 años (19,4%); no mostró diferencias estadísticamente significativas, con un p = 0,436 (Tabla 1). Al evaluar la depresión, se encontró depresión moderada en el grupo etario de 19 a 22 años (11,1%), y ausencia de depresión en el 56,5% del total de la muestra. No se hallaron diferencias estadísticamente significativas p = 0,715 (Tabla 2).

**Tabla 1:** Relación del grado de estrés (SISCO) según edad

Escala SISCO	Grupo etario			Total	Valor p
	19-22	23-25	Más de 26		
Bajo	20 (18,5%)	16 (14,8%)	5 (4,6%)	41 (38%)	0,436*
Medio	10 (9,3%)	8 (7,4%)	8 (7,4%)	26 (24,1%)	
Alto	21 (19,4%)	13 (12%)	7 (6,5%)	41 (38,0%)	
Total	51 (47,2%)	37 (34,3%)	20 (18,5%)	108 (100%)	

*Prueba exacta de Fisher

**Tabla 2:** Relación del grado de depresión (Beck) según edad

Escala Beck	Grupo etario			Total	Valor p
	19-22	23-25	Más de 26		
Normal	25 (23,1%)	24 (22,2%)	12 (11,1%)	61 (56,5%)	0,715*
Leve	12 (11,1%)	7 (6,5%)	4 (3,7%)	23 (21,3%)	
Moderado	12 (11,1%)	6 (5,6%)	3 (2,8%)	21 (19,4%)	
Extremo	2 (1,9%)	0 (0%)	1 (0,9%)	3 (2,8%)	
Total	51 (47,2%)	37 (34,3%)	20 (18,5%)	108 (100%)	

*Prueba exacto de Fisher

Al evaluar la relación de estrés (escala SISCO) y el ciclo de estudios, se encontró estrés alto (10,2%) en los estudiantes que cursan el VII ciclo; no mostró diferencias estadísticamente significativas, con un p = 0,194 (Tabla 3). Al evaluar la depresión, se encontró depresión leve en el 5,6% de los estudiantes que cursan el VI ciclo, y ausencia de depresión en el 56,5%. No se encontraron diferencias estadísticamente significativas p = 0,679 (Tabla 4).

**Tabla 3:** Relación del grado de estrés (SISCO) según ciclo

Escala SISCO	Ciclo de estudio					Total	Valor p*
	Ciclo VI	Ciclo VII	Ciclo VIII	Ciclo IX	Ciclo X		
Bajo	16 (14,8%)	8 (7,4%)	4 (3,7%)	6 (5,6%)	7 (6,5%)	41(38%)	0,194*
Medio	3 (2,8%)	6 (5,6%)	4 (3,7%)	5 (4,6%)	8 (7,4%)	26(24%)	
Alto	7 (6,5%)	8 (7,4%)	11 (10,2%)	7 (6,5%)	8 (7,4%)	41(48%)	
Total	26(24,1%)	22(20,4%)	19(17,5%)	18(16,7%)	23(21,3%)	108(100%)	

*Prueba exacto de Fisher

**Tabla 4:** Relación del grado de depresión (Beck) según ciclo

Escala Beck	Ciclo de estudio					Total	Valor p
	Ciclo VI	Ciclo VII	Ciclo VIII	Ciclo IX	Ciclo X		
Normal	15 (13,8%)	15 (13,8%)	8 (7,4%)	9 (8,3%)	14 (13,0%)	61(56,5%)	0,679*
Leve	6 (5,6%)	4 (3,7%)	3 (2,8%)	5 (4,6%)	5 (4,6%)	23(21,3%)	
Moderado	5 (4,6%)	1 (0,9%)	7 (6,5%)	3 (2,8%)	5 (4,6%)	21(19,4%)	
Extremo	0 (0%)	1 (0,9%)	1 (0,9%)	1 (0,9%)	0 (0%)	3(2,8%)	
Total	26 (24%)	21 (20,3%)	19(17,6%)	18(16,6%)	24(22,2%)	108(100%)	

*Prueba exacto de Fisher

Al evaluar el estrés y la depresión en los estudiantes del VI al X ciclo de la Carrera de Odontología de la Universidad Científica del Sur, se halló que la depresión moderada con estrés alto representó un 18,5% del total, y sin depresión y estrés bajo, un 31,5%, con diferencias estadísticamente significativas: p < 0,001 (Tabla 5).

**Tabla 5:** Relación entre estrés y depresión en estudiantes de la carrera de Odontología

Escala Beck	Escala SISCO			Total	p
	Bajo	Medio	Alto		
Normal	34 (31,5%)	17(15,7%)	10 (9,2%)	61 (56,4%)	<0,001
Leve	7 (6,5%)	8 (7,4%)	8 (7,4%)	23 (21,3%)	
Moderado	0 (0%)	1 (0,9%)	20 (18,5%)	21 (19,4%)	
Extrema	0 (0%)	0 (0%)	3 (2,8%)	3 (2,8%)	
Total	41 (38%)	26 (24%)	41 (37,9%)	108(100%)	

*Prueba exacta de Fisher

## DISCUSIÓN

El estrés y la depresión en estudiantes de ciencias de la salud y otras carreras universitarias se viene percibiendo desde hace varias décadas, y suelen estar asociados con factores académicos, psicosociales, laborales y económicos ^(11, 12)^. Algunos estudios señalan que, en la carrera de Odontología, el porcentaje de estrés académico varía entre un 38 y un 40%, y se relaciona con la alta carga académica, los excesivos trabajos o tareas para la casa, los exámenes, el miedo a reprobar y las prolongadas horas de estudio semanal ^12^. Hoy en día, el estrés se ha convertido en un problema a nivel mundial que genera grandes problemas psicológicos e incrementa la tasa de mortalidad incluso en países desarrollados ^(13, 14)^. Esto propicia el inicio de enfermedades sistémicas en los estudiantes. Por ello, resulta importante prestar atención a los síntomas y los cambios emocionales que puede presentar el estudiante universitario, ya que esto puede afectar su desempeño académico, sobre todo ante la crisis sanitaria que venimos atravesando por la COVID-19 ^(2, 15)^. El cambio en la modalidad de estudios que los estudiantes de odontología experimentan les causa una gran frustración ^16^.

En el 2018, se realizó un estudio sobre una universidad privada de Lima con 140 participantes, quienes fueron evaluados mediante el cuestionario Dental Environment Stress Questionaire, tomando en consideración el estrés, la edad, el sexo y el ciclo que cursaban. Se obtuvo como resultado que el 75,7% de los participantes padecía de estrés académico, con una relación significativa entre el ciclo y la edad del participante que repercutía más en los ciclos preclínicos y en los jóvenes más que en los adultos ^17^. Al comparar esos resultados con los del presente estudio, se halló que todos los participantes tenían estrés académico, pero en este caso no se encontró una relación significativa entre la edad y ciclo. Podemos afirmar que el estrés académico se presentó más en la población de 19 a 22 años y que el estrés es más frecuente en los estudiantes más jóvenes. En relación con el estrés académico y el ciclo no se pudo encontrar una relación ya que solo se tomó la muestra del 6.^o^ al 10.^o^ ciclo, que corresponde a cursos clínicos; pese a ello, se evidenció una mayor carga de estrés en el 7.^o^ ciclo.

En el ámbito científico internacional, en el 2016, se realizó un estudio en estudiantes de medicina de la Universidad de Fayoum, en el cual se evaluó a los estudiantes mediante la escala Depression, Anxiety and Stress Scale-21 (DASS-21). La prevalencia de estrés fue del 62,4%; la de ansiedad, del 64,3%; y la de depresión, del 60,8% en los participantes. Los puntajes más altos de estrés y ansiedad estaban asociados con el sexo femenino y la edad avanzada ^18^. En el caso del presente estudio, fue realizado en estudiantes de Odontología y estos fueron evaluados mediante la escala de SISCO para estrés académico y la escala de Beck para depresión. El nivel de estrés fue mayor en dicha población, ya que todos los estudiantes lo padecían, ya sea en un nivel bajo, medio o alto; sin embargo, el 100% de la muestra presentaba depresión y el 47,2% de esta afección se presentó en estudiantes 19 a 22 años. 

En el 2020, se realizó un estudio transversal en Arabia Saudita con la finalidad de evaluar el impacto psicológico de los estudiantes de Odontología utilizando el cuestionario Depression, Anxiety and stress scale-21, mediante el cual se encontró que el 60,6% de los estudiantes tenía depresión; el 37%, ansiedad; y 34,9%, estrés ^19^. En el presente estudio, el 46,5% tuvo depresión y el 37,9%, estrés académico alto. Si estos resultados los comparamos con el estudio realizado en el 2016 ^18^, podemos evidenciar que la tasa de depresión entre los estudiantes de Odontología y Medicina no ha tenido diferencias significativas; sin embargo, si lo comparamos con el estudio en una universidad peruana del 2018, podemos evidenciar que el porcentaje de estrés en los estudiantes fue del 75,7%.

El presente estudio tuvo como principal limitante la cantidad de alumnos participantes; al no alcanzar la muestra ideal, se procedió a incluir en el estudio a estudiantes del VI cuatrimestre. La muestra se tuvo que dividir en intervalos por conveniencia en la sección edad para que no varíe el porcentaje de afectados y no existan sesgos en la medición y los resultados. Este estudio tiene una gran importancia académica para cada estudiante y docente de la carrera; ya que al saber que existe una tasa considerable de estrés y depresión se pueden aplicar estrategias para contrarrestar su incidencia a través de clases más didácticas, un servicio de psicología y orientación para que los estudiantes sepan cómo controlar el estrés y la incertidumbre por la excesiva carga académica y la nueva modalidad de estudio que afronta la educación en el Perú, al ser 100% virtual. El apoyo profesional adecuado permitirá adecuar las estrategias específicas para minimizar o incluso erradicar el estrés propiciando el equilibrio y organización de las obligaciones.

## CONCLUSIONES

Existen relación entre el estrés académico y la depresión en los estudiantes del 6.^o^ al 10.^o^ ciclo de la carrera de Odontología de la Universidad Científica del Sur. No se encontró relación entre el nivel de depresión y de estrés con la edad y el ciclo de estudio de los estudiantes.

## Fuente de financiamiento:

Autofinanciado
